# A qualitative research of adolescents with behavioral problems about their experience in a dialectical behavior therapy skills training group

**DOI:** 10.1186/s12888-020-02649-2

**Published:** 2020-05-19

**Authors:** Eva Sesma Pardo, Aránzazu Fernández Rivas, Pablo Orgaz Barnier, Marina Beá Mirabent, Iñaki Kerexeta Lizeaga, Aída Díaz Cosgaya, Ana Catalán Alcántara, Esther Vivanco González, Blaise Aguirre, Miguel Angel González Torres

**Affiliations:** 1 Psychiatry Service, Basurto University Hospital, Avda Montevideo, 18 48013 Bilbao, Spain; 2 Department of Neuroscience, University of the Basque Country, Bilbao, Spain; 3 East Residential. McLean Hospital, Belmont, USA; 4 Assistant Professor of Psychiatry. Harvard Medical School, Boston, USA

**Keywords:** Adolescents, Dialectical behavior therapy, Emotional dysregulation, Qualitative research, Focus groups

## Abstract

**Background:**

Several quantitative studies support the effectiveness of the Dialectical Behavior Therapy (DBT) psychosocial skills training group component for adolescents with impulse-control disorder and/or emotional dysregulation. However, qualitative research to assess this psychotherapeutic tool in the adolescent population is sparse. This study aims to examine the subjective experience of adolescents with behavioral issues who have completed DBT skills training group, as well as using this experience to extract hypotheses regarding its usefulness which can then be verified at a later time by means of quantitative instruments.

**Methods:**

We developed a qualitative study by using focus groups with adolescents (*N* = 20) whose diagnosis includes symptoms such as behavior disorder, impulse-control disorder and/or emotional dysregulation, and good informants, who have completed DBT skills training. Three focus groups were created.

**Results:**

The subjective experience of adolescents who have completed a DBT skills training group is collected in four main categories: experience of illness, motivation for therapy, experience of therapy and results of the therapy.

**Conclusions:**

Adolescents with behavioral problems assess their participation in the DBT skills training group positively, even recommending its usefulness to healthy population. Beyond learning skills, they emphasize the intrapsychic changes (as improvement in reflective activity) that they objectify after the group experience.

## Background

Adolescence can be seen as a period of coping with a variety of challenges, necessary for normal development [1]. During adolescence, emotional dysregulation leading to impulsivity and the emergence of behavioral problems is common [2]. Emotional dysregulation consists of poor control over his or her own affective expression in different situations. It is characterized by little flexibility and spontaneity, lack of control and disruptive behaviors. Following Linehan’s biosocial model, we conceptualize emotion dysregulation in borderline personality disorder (BPD) as consisting of four components: emotion sensitivity, heightened and labile negative affect, a deficit of appropriate regulation strategies, and a surplus of maladaptive regulation strategies. Emotional dysregulation is a dimensional entity, not exclusively related to any specific disorder and may be present both in externalizing and internalizing disorders [3–5].

Dialectical behavior therapy (DBT) was developed specifically to address Borderline Personality Disorder (BPD). It includes four components: individual therapy, psychosocial skills training, telephone coaching for patients, and a supervision team for the therapists [6, 7]. DBT is conceptualized as a transdiagnostic treatment [8], the aim of which is to help the patient initiate functional behavior, even when they are experiencing very intense emotions [9]. Skills training appears to be the most effective component of DBT when emotional dysregulation predominates [10] and also for adolescent patients [11–13]. It includes five modules (mindfulness, distress tolerance - DT, emotion regulation - ER, interpersonal effectiveness -IE, and walking the middle path) taught throughout sixteen weeks in group format. Mindfulness and DT are the skills most valued by adolescents [14, 15].

Several quantitative studies support the effectiveness of DBT for adolescent populations who present multiple problems [16–19] including BPD traits, suicidal and/or self-injurious ideation and behavior [20–25]. Furthermore, preliminary effectiveness of DBT for pre-adolescent children with mood disruptive dysregulation disorder has also been reported in a randomized controlled trial [26].

The subjective view of young adults treated with DBT regarding the process and outcome of the therapy has been explored using qualitative methodology. Factors such as therapeutic relationship, self-motivation to change, validation and the perception of genuine interest in the support offered by the leaders of the skills group, are described as fundamental components to the improvement experienced [27–29]. The most valued skills were mindfulness and DT [30].

Even though DBT for adolescents has provided consistent evidence of efficacy, there are often patients who drop out, others who show insufficient outcomes and some others who find difficulties in adapting to the requirements of participation in skills groups. We think the exploration of the subjective experience of participants with a qualitative methodology could provide potentially interesting insights regarding ways to deal with those problems. Besides that, we believe the qualitative exploration of subjective experience in a group context could not only add to the existing knowledge about emotional dysregulation and interpersonal difficulties shown by those patients but could also give us clues to a better understanding of the healing process itself.

We conducted a study employing qualitative methods with the following objectives: (i) To better understand the subjective experience of adolescents with behavioral problems who have participated in DBT skills training groups, (ii) To assess the perceived usefulness of those skills, and (iii) To ascertain the subjective benefit from participating in a group as a place of learning with peers and authority figures.

## Method

Qualitative methodology is essential for health sciences when studying aspects unapproachable by other methodologies. These elements include values, attitudes, expectations, and the impact of suffering and sociocultural factors that influence health and illness. Within those methods of a qualitative nature, we chose focus groups due to the advantages they offer [31]. The key data generated by focus groups is the narratives of those participating [32–34]. The research team for our current study was composed of clinicians with experience in the field of psychotherapy and qualitative research [35].

### Selection of the sample

Skills training groups for adolescents have been conducted by the Psychiatry Service of Basurto University Hospital (Bilbao), since January 2010. In parallel, their parents and families were offered similar groups. Adolescents who completed the skills training group (16 weekly sessions) and had shown during the process sufficient capacity to articulate opinions and disposition to share them, were considered as “good informants”. All those who met inclusion criteria (Table 1) were contacted by telephone and invited to participate. Their participation was voluntary and they received no incentive.

**Table 1:** Inclusion and exlusion criteria

INCLUSION CRITERIA	EXCLUSION CRITERIA
• *Age between 14 and 17 years*	• *Clinical contraindication to participate in a group at the time of the study*
• *Diagnosis that includes behavioural disorder, impulsivity and/or emotional dysregulation as a symptom*	• *Inability to provide informed consent*
• *Having completed a skills training group*	• *Not being a good informant*
• *Informed consent signed by the adolescent and parent or legal guardian*	

We contacted 30 patients of which 24 agreed to participate. After parents and adolescents signed informed consent, they were enrolled and allocated to different focus groups. Of the 24 enrolled, four candidates did not attend the focus group providing no reason or explanation. We performed three focus groups with 20 participants. In focus group number 1, 7 participants were cited and 6 attended. In focus group number 2, 7 participants were cited and 6 attended. In focus group 3, 10 participants were cited and 8 of them attended.

The sample consisted of 20 adolescents (mean age 15.40; SD 1.39). Sociodemographic and clinical data are shown in Table 2. All assessments and diagnoses were performed by a senior Child and Adolescent psychiatrist using DSM-IV-TR [36] prior to inclusion in the skills training group.

**Table 2:** Profile of sample

	***Number***	***Percentage***
***Gender***		
Female	16	80%
***Living with***		
Biological parents	18	90%
Adoptive parents	1	5%
Other family members	1	5%
***Occupation***		
Academic	20	100%
***Axis I diagnosis***		
Adjustment disorder with mixed disturbance of emotions and conduct	13	65%
Unspecified impulse control disorder	3	15%
Conduct disorder	1	5%
Unspecified disruptive behaviour disorder	1	5%
Adjustment disorder with disturbance of conduct	1	5%
Intermittent explosive disorder	1	5%
***Axis II diagnosis***		
Personality disorder	15	75%
Borderline personality features	2	10%
***History of admission to psychiatric unit***		
Yes	16	80%

### Focus groups and data collection

The research team had previously met before starting the focus groups to prepare a script delineating the scope of inquiry. We agreed upon a set of questions covering topics that in our view merited initial exploration. Two weeks earlier, we piloted these questions with two individuals who had met inclusion criteria in order to establish the utility of the questions as well as to identify unconsidered topics. After determining that the question-set would provide insightful commentary, the questions were put forward to the entire focus group. After completing the first focus group, two researchers independently studied the videotapes and transcripts. Then, all the researchers agreed on adapting the initial questioning path integrating the new ideas expressed in the first group. This process was also repeated after the second group. After completing the third focus group, it was considered that information saturation (no new information added) had been achieved. Groups of ninety minutes duration were performed and were led by two researchers unknown to the participants. A trusting and empathic environment was actively promoted [35].

### Data analysis

**Fig. 1:**
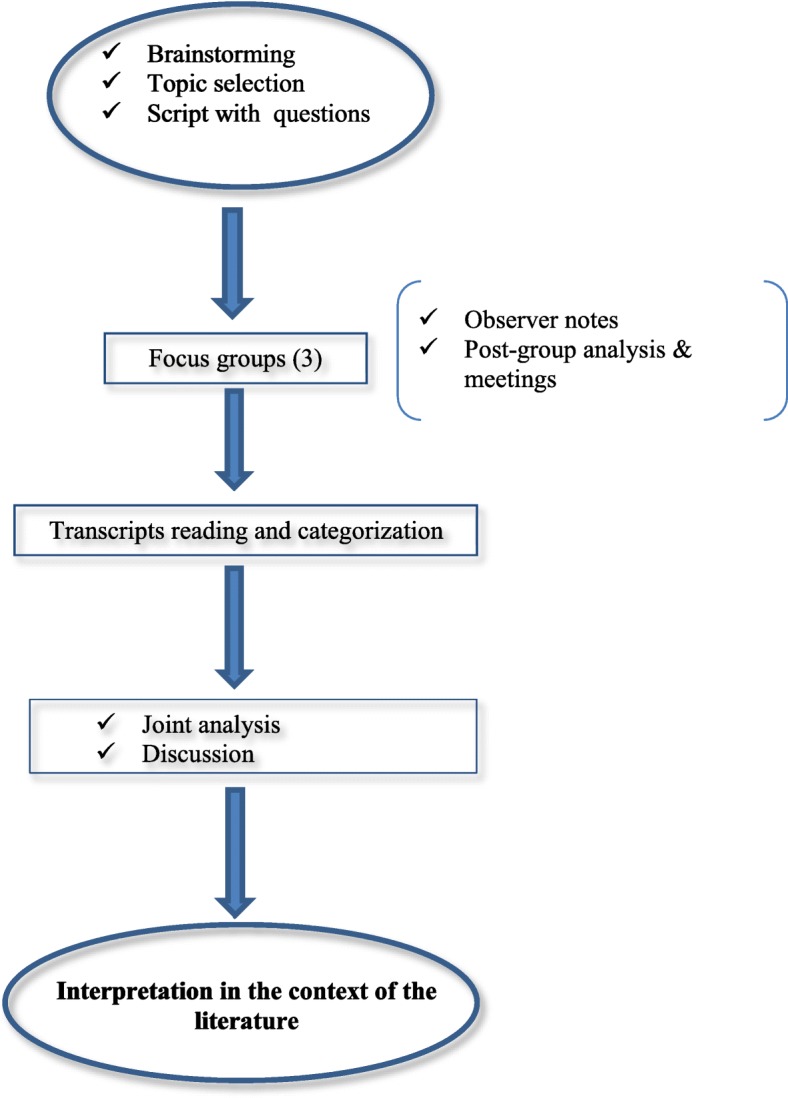
Outline of data analysis process

The data analysis process is shown in Fig. 1. The transcript documents were the primary data source. Each of the researchers first performed an in-depth independent reading and then proceeded to the categorization stage. Research team met twice a week to share their findings and reviewed disagreements and discrepancies until a final consensus regarding categorization was reached.

Analysis of the transcriptions, as required by qualitative methodology (exploratory, inductive and ethnographic), was based on Grounded Theory [32] .The discourse is codified, broken down into units of meaning, and categorized by a procedure of progressive abstraction from a textual level to a conceptual level by applying the constant comparative method. For the qualitative analysis of the data, we used the MAXQDA software program (version 12) [37].

## Results

Ideas expressed by the participants were grouped into four categories: experience of illness, motivation for therapy, experience of therapy, and results of therapy (Table 2).

### Experience of illness

#### Emotional dysregulation

Adolescents reported the difficulties they had in regulating emotions as a core issue. The blocking of communication or the failure of a mechanism such as emotional restraint, may later lead to the appearance of mood disorders and behavioral dyscontrol.

*“And what I did was try to keep it in, keep it in (the distress) and … it worked until I burst”* (A; Male, 23).

#### Behavioral dyscontrol

They describe behavioral dyscontrol, with an aggressive undertone, as a way of regulating emotions.

*“she (mother) didn’t do anything wrong, but for the most trivial reasons, like if she would disagree with me, I’d hit her to vent my anger”* (A; female, 16).

#### Identity issues

Adolescents describe identity issues in relation to poor or devalued self-concept, and perceive the identity of sufferer as immutable.

*“Well I was a person who … let’s say … lived for her illness … let’s say I thought I was my own illness and I don’t know, I didn’t want to change”* (M; female, 19).

#### Global functional deterioration

They observe how their symptoms have a direct impact on their global functional behavior, describing it as a vital break with a pre-existing global functional deterioration.

*“I quit going to class for a year, broke up with the boyfriend I had for a long time, stopped going out with my best friends … it was awful with my family … so yeah, there were repercussions”* (A; female, 17).

#### Environmental feedback

The environment responds to the problems of adolescents in various ways. Sometimes it can act as an aid by offering support and external containment,

*“The thing is, in my case, my parents have always been there for me, they’ve been the ones who’ve helped me in everything, I’ve never had any kind of problem with my parents … ”* (Y; male, 17).

however, in other cases it can make problems worse, in invalidating environments or where the stigma attached to mental illness is manifestly present.

*“They’ve even called me a mental bitch, crazy … all sorts of names”* (E; female, 17).

### Motivation for therapy

#### Time factor

Adolescents state that motivation for therapy varies depending on the moment of the illness course.

*“I think it (the therapy) came at just the right time and … maybe what I was referring to was that … this was the beginning of realizing something was wrong with me, I don’t know how to explain it, this helped me realize”* (M; female, 19).

#### Group attendance

It is suggested that group attendance may represent an objective parameter to assess motivation for change and active involvement in therapy.

*“I only missed once (one group session) and it was ‘cause I had my tonsils removed, I wanted to change and took it very seriously”* (E; female, 17).

#### Resistance to change

Resistance to change and consequently to receive help at some point during the course of their illness is another characteristic shared by adolescents.

*“and well, I didn’t think I wanted to change, nor I felt comfortable or with the strength to change, I didn’t want no help, it’s happened to all of us at some point”* (T; female, 21).

#### Self-motivation for treatment

The importance of self-motivation for change is stressed as an essential factor for the effectiveness of therapy. Without it, the therapy does not work.

*“You know, if a person doesn’t want to change, they’ll never change, like, even if someone goes to lots of groups, lots of therapies or even if they travel to 50 different countries to do different therapies, that doesn’t matter, if someone isn’t willing to change, they won’t change”* (L; female, 17).

### Experience of therapy

#### Group frame

Those participating in this study mention certain positive aspects within the group space such as being psychopathologically heterogeneous groups,

*“No one else (in the therapy) had my same problem and I think this was good, cause if I’d found people who were as angry as I was at the world … it wouldn’t have done me no good”* (A; male, 22).

and the participatory approach as a differentiating characteristic from other therapies.

*“(the therapist) for example he made us go up to the blackboard, to explain it, participate … it was like … he made us study the theory, to put it some way, and then would ask us to come up and explain it … , it was different from other groups”* (N; female, 18).

The adolescents see some aspects of the group space as therapeutic in themselves. For example, they define the group space as a validating space where there is no room for stigma

*“Precisely when I was at my worst I thought I wanted to have someone to talk to who would understand me, so I could share it, that was enough to calm me down”* (L; female, 17).

and where interpersonal relationships are encouraged, forming group cohesion.

*“And there was a boy (in the group) who was a metalhead, he was great, we would laugh so much … and that would also help me to get better, that good relationship … ”* (T; female, 21).

*“Well … I don’t know, both of us helped each other to get better and get out of the hell we were in and all”* (I; male, 20).

#### Content

Those participating in this study point out the importance of the images and graphics to help understand the skills,

*“I remember the circles, which was rational (mind), irrational or something like that”* (A; female, 17).

*“I think that one about the emotional, rational (mind) … wise mind, I think that’s what stuck with me the most ‘cause it was an image … you know, it was plain simple”* (A; female, 16).

which, in turn, coincides with the importance given to mindfulness, as mindfulness and DT are the most valued skills.

*“But that thing about the wise mind and the emotional mind did stick with me”* (L; female, 17).

*“I will remember this lesson all my life, I use it a lot, it was an exercise which said suffering equals pain plus lack of acceptance, and when you accept that pain, it’s no longer suffering and only the pain remains, this has helped me greatly”* (A; female, 18).

However, they do not associate the usefulness of homework with the generalization of the skills learned in the group as the therapists instructed them.

*“Yeah, I always (did the homework), ‘cause I felt it was an obligation, I’m not sure if it helped me a lot but I did do it”* (L; female, 17).

It is suggested that skills implementation should be done unconsciously, in situations without a high emotional load, where acting with a wise mind is easier.

*“Yeah, that’s it, since the beginning what he (the therapist) taught us was what to do in situations like that, so I would always try to put it to the test, I even got the point where I could do it without thinking”* (S; female, 15).

*“If the anger, let’s say, wasn’t of a very high level, I could do it (apply the skills) without realizing it”* (O; female, 15).

Nevertheless, if the situation poses a high emotional load then the implementation of the skills has to be done consciously, with total awareness, since acting with a wise mind is more difficult.

*“Well, I remember one time I was in Mathematics class with the teacher I loved so much (sarcasm). I didn’t' want to argue with him in class again so I started to shriek “come on wise mind, wise mind” everyone was freaking out while I kept on “wise mind, wise mind” so I wouldn’t swear at him, ‘cause otherwise I would disrespect him like I always did”* (N; female, 15).

#### Relationship with peers

Adolescents observe that the relationship with peers in the skills groups serves as a measuring tool to assess their own severity.

*“Compared to the other girls I was with (in the group), my problems didn’t seem that bad”* (S; female, 15).

and point out that the phenomenon of universalization is a relief factor at the beginning of therapy.

*“I realized there were more people (in the group) other than me who were ill, and, let’s say that doesn’t make you feel so lonely … ”* (M; female, 19).

#### Relationship with therapists

Regarding the relationship with therapists, emphasis is placed on the need for their presence in order to regulate communication among the peers in the groups,

*“I left the (WhatsApp) group chat … in any group of people with problems there has to be a therapist in between. Always”* (L; female, 17).

and they value the therapist’s genuine interest to help as a crucial quality that contributes to their improvement.

*“they (the therapists) would motivate you a lot, I remember I didn’t do the homework the first time I came here ‘cause I wasn’t motivated at all. Then the second time when I did it, he (the therapist) congratulated me, when I got the stuff done, when I did it well”* (N; female, 18).

*“they worried about what things served us the most to get better in the future, I don’t know, that was it in general, they were really implicated … ”* (A; female, 17).

### Results of the therapy

#### Positive assessment

Adolescents assessed the skills training group (DBT) positively and recommended its potential usefulness in a healthy population.

*“Well, I thought it was all very interesting and that’s something which is supposed to be basic but I think it should be way more in people’s minds, not only in our case; people who have problems, but in everyone else’s”* (A; female, 17).

They have a realistic view of the results, being aware of the reversibility of the change, the results become noticeable over time and, likewise, last for a while.

*“and eventually, as time went by (in the therapy) I did end up feeling an improvement”* (O; female 15).

*“yeah, but … even though, I think, to me, considering what I’m seeing lately, the group has served me just for some time ‘cause now I’m beginning to feel bad again”* (I; male, 20).

The results are not idealized. Participants describe partial improvement and point out how difficulties in generalizing skills in certain situations still persist, especially those with a high emotional load.

*“yeah, mine (the illness) stopped some time ago but I’m still having some bad periods … ”* (M; female, 19).

The adolescents state that for the skills to work, motivation for change is essential, where life goals and self-reinforcement come into play.

*“So I think all of us have experienced that what really makes us change is that we want to change, cause no one can do nothing for us, we have to take that kind of initiative like, alright, I’m gonna get better … ”* (A; female, 16).

#### Acquiring abilities

The participants state that they acquire behavioral ability in the DBT skills training group to more adaptively cope with crisis situations and use tools to understand their own suffering. This on turn leads them to become aware of the difficulties they present and provide them with an understanding about their illness,

*“I think (the therapy) came at the perfect time and … what I meant was that … this was the beginning of realizing something was wrong with me, I don’t know how to explain it … this helped to … stop seeing everything in black and white and lose control”* (M; female, 19).

*“to also make me realize that one can’t make a big deal out of everything (when asked, How did the therapy help you?)”* (A; female, 16).

#### Intrapsychic changes

In parallel, intrapsychic changes occur, such as improvements in reflective activity. Projection is replaced by reflection, which allows patients to theorize about their illness from the observing self and to give meaning to their previous experience of suffering.

*“every time I felt I wasn’t welcomed cause I had done something bad … to stop feeling upset, sometimes I would take two or three pills so I could swallow them … so I could feel as if I was swallowing my pain or something, to find something which would hurt me physically and not psychologically”* (N; female, 15).

*“cause you end up harming yourself … to forget the psychological pain through physical pain”* (M; female, 19).

## Discussion

Adolescents in the skills training groups become aware of their problems and start to reflect upon causative mechanisms. They report difficulties in regulating their emotions as a core issue [3, 5, 6]. Following the group experience and the improvement in reflective function, they realize self-injury behaviors have an anxiolytic purpose [4].

The function our adolescents give to behavior coincides with the existing published studies. They use behavior to externalize internal difficulties [30]. This is a fact that supports the consideration of emotional dysregulation as a “transdiagnostic process” present in both internalizing and externalizing disorders [6].

Regarding their general experience of the skills training groups, participants mention several positive aspects:

They point out two qualities that are therapeutic in themselves: a validating environment and group cohesion. The group is referred to be validating when it is interested in and understanding of the individual’s experience and encourages them to express and communicate their emotions. Group cohesion provides relief, from the beginning of therapy, thanks to the universalization phenomenon and differentiation from peers [38]. However, we cannot consider these qualities as unique to DBT, because they can happen in other group approaches.

Adolescents also state a direct relationship between therapy effectiveness, self-motivation for change, and an active involvement in treatment [31, 34]. Participants highlight how the benefits perceived are only obtained when the person is motivated for therapy, connecting this issue with the importance that DBT gives to the motivational work.

The participants express their preference towards the acceptance skills, mindfulness and DT, a finding consistent with existing literature [6, 17–19]. They report that during therapy, mindfulness enables them to realize what is happening to them. ER skills allow them to rethink situations of the past and help to manage difficulties in the present. IE skills help them to have a representation of themselves and the other and be more effective in interpersonal relationships. These skills with the basic ingredients of DBT, validation and dialectics, favor intrapsychic changes such as an improvement in reflective activity.

Participants also express several critical views about the experience:

They do not seem to understand the relevance of homework assignments and how they promote the generalization of skills. It is true that we have not encountered rejection from the adolescents in the study towards completing the homework. This could be a topic to be more thoroughly addressed in groups of adolescents, making them truly aware of the importance of this aspect of the treatment to extend the effect of training across all areas of their lives.

Another relevant criticism concerns their view that improvement may not be complete or permanent, or that it does not come immediately, and relapses are possible. They see skills memorization and application with complete awareness as something difficult during an episode of high emotional dysregulation. Following these thoughts, some degree of maintenance or continuity of the treatment can be considered as a clear need. This could take the form of an extended period of individual DBT sessions, the so called “booster sessions” or graduate groups (involving participants who have already completed a skills training group) [12]. We opt for using a combination of individual sessions plus graduate groups as an ideal maintenance strategy. In any case the goals are relapse prevention, generalization of skills and promotion of behaviors that induce a positive quality of life.

Participants also mention other interesting proposals.

Taking their narratives into account, skills training is perceived as useful for emotional dysregulation issues. They regard its potential helpfulness also in healthy individuals, a finding shown in a qualitative study on adult population [27]. Including skills training in the socio-emotional learning curriculum for adolescents would equip them with tools to cope with different life situations, increasing their strength and consequently their resilience [38]. Likewise, this training may be of great help to encourage treatment adherence in those adolescents who not only suffer from a chronic medical illness [39] but also have a high resistance to assume and exercise responsibility for the treatment they require.

Therefore, our findings suggest that the flexibility and high structuring of skills training allow this component of DBT to become a vehicle through which we can reach out to adolescents and the multiple areas of their life.

In light of these findings, we see several avenues open to future studies.

Given the difficulties adolescents encounter to keep the gains obtained after the treatment, there is a clear need to evaluate maintenance interventions.

The important programs currently deployed to apply these interventions to healthy adolescents should be thoroughly extended and studied to examine their contributions to normal development and resilience [40].

Finally, as a logical step continuing from our study, subjective experience of parents and families of adolescent patients, participating in the parents skills training groups, should also be explored. In this instance, we believe a qualitative methodology is very well suited to carry on this study.

### Limitations

Our study has two kinds of limitations. One is related to qualitative methodology itself and other specifically related to our study.

Qualitative studies all have an inherent problem of generalizability because the samples do not usually represent the population from where it comes. It is important to note that the goal here is to capture existing subjective experiences without assigning to them frequencies or intensities. These will be later studied through quantitative designs. The focus group format provides some important advantages but also implies limitations as the interaction among patients can favor inhibition of more passive individuals whose opinions fade to the background as a result of those who express more prominently their own opinions. Finally, we must not forget that discou-rse on any aspect of reality is conditioned by the semantic context in which an individual finds himself and in which he has grown up.

Some specific limitations of our study can also be pointed out. There is a lack of information about those subjects who declined to participate after having signed the informed consent. A possibility is that perhaps they had a negative view of the program and felt they would not be able to successfully voice their opinions and could even face rejection. Additionally, the researchers conducting the focus groups had never met the participants before the study, but the adolescents knew they belonged to the staff and this could have influenced the open sharing of their views.

## Conclusions

Adolescents with behavioral problems who participated in this study identify the difficulties they have in adaptively managing the emotions that they feel to be a core issue. After completing skills training, they assess the psychotherapeutic tool positively and recommend its usefulness to a healthy population. They report that the motivation for change and the time factor are two aspects closely related to the effectiveness of the therapy. Furthermore, they also note that the most valued skills are mindfulness and DT. With regard to the results of the therapy, in addition to acquiring skills to adaptively manage suffering, they describe intrapsychic changes, such as an improvement in their reflective ability. This allows them to theorize about the difficulties they faced in their past and to better understand themselves in the present.

## Data Availability

Transcriptions are kept by the authors. Interested researchers can access them contacting the corresponding author and after complete anonimisation of participants is secured.

## Abbreviations

DBT: Dialectical Behavior Therapy
BPD: Borderline Personality Disorder
DT: Distress Tolerance
ER: Emotion Regulation
IE: Interpersonal Effectiveness
DSM-IV-TR: Diagnostic and Statistical Manual of Mental Disorders – IV - revised text
AEPNYA: Spanish Association of Child and Adolescent Psychiatry
